# Food restriction during pregnancy and female offspring fertility: adverse effects of reprogrammed reproductive lifespan

**DOI:** 10.1186/s13048-017-0372-x

**Published:** 2017-12-28

**Authors:** Abdel Halim Harrath, Abdulkarem Alrezaki, Lamjed Mansour, Saleh H. Alwasel, Stefano Palomba

**Affiliations:** 1 Zoology Department, College of Science, King Saud University, Riyadh, Saudi Arabia; 2 Unit of Reproductive and Developmental Biology, Faculty of Science of Tunis, University of Tunis El Manar, Tunis, Tunisia; 3 Unit of Gynecology and Obstetrics, Grande Ospedale Metropolitano “Bianchi – Melacrino – Morelli”, Reggio Calabria, Italy

**Keywords:** Follicle, Food restriction, Ovary, Oocyte, Pregnancy

## Abstract

**Background:**

Food restriction during pregnancy can influence the health of the offspring during the adulthood. The aim of the present study was to examine the effect of maternal food restriction (MFR) on the reproductive performance in female rat offspring from the first (FR1) and second (FR2) generations.

**Methods:**

Adult virgin Wistar female rats were given free access to tap water and were fed *ad libitum* on standard rodent chow, were mated with virgin adult males, and then were randomly divided into two groups: controls (that was fed *ad libitum* *)* and food-restricted group (FR, that was given only 50% of *ad libitum* food throughout gestation). Their first (FR1) and the second (FR2) generation of offspring were fed *ad libitum* and sacrificed before puberty and at adulthood. Their ovaries were removed and their histology evaluated by estimating the number of follicles (total and at various stages of folliculogenesis), and the presence of multi-nuclei oocytes and multi-oocyte follicles.

**Results:**

Total number of ovarian follicles was lower in FR1 females at week 4 in comparison with controls, while it was not different in FR2 females vs. controls. The number of the primordial follicle was lower in FR1 and FR2 females vs. controls at both week 4 and at week 8. When compared to the controls, the follicles containing multi-nuclei oocytes were more frequent in ovaries from FR1 and FR2 females at week 4, and higher and lower respectively in ovaries form FR1 and FR2 females at week 8.

**Conclusion:**

MFR affects ovarian histology by inducing the development of abnormal follicles in the ovaries in first and second generation offspring. This finding could influence the ovarian function resulting in an early pubertal onset and an early decline in reproductive lifespan.

## Background

Maternal nutritional status during gestation is a key determinant for the health and physiology of the offspring at adulthood, and that influence is mainly established during early development, i.e. well before birth [1–5].

Many adulthood diseases can be linked to the environment within which the embryo has developed, including abnormal nutritional, environmental, and hormonal insults that may have changed the developmental trajectory of the fetus [1, 2]. According to this hypothesis, the origins of common diseases may be due to the environment that the fetus directly senses via the mother [2, 5–8]. In particular, maternal food restriction (MFR) has been associated with coronary heart disease and increased arterial blood pressure [2], reduced nephron endowment and increased renal morbidity in adulthood [9], and may affect physical growth and neurobehavior in newborns [10]. Malnutrition during gestation has been associated with hepatic steatosis, type 2 diabetes, and obesity during adulthood [5, 11–15]. During late gestation, MFR is associated with metabolic signaling dysfunction in the liver, and predisposes the offspring to insulin resistance [5].

Oocyte quality is a critical determinant for the developmental trajectory of the fetus [16, 17]. Many forms of female reproductive disruptions have been linked to the prenatal environment, and it is likely related to early oocyte formation, which is vulnerable to numerous environmental effects [4]. In comparison with girls who were born appropriate for the gestational age, girls born small for the gestational age have a reduced reproductive lifespan, indicated by a decrease in ovarian size that is associated with low ovulation rates [18, 19], advanced menarche, and early menopause [20–22]. Polycystic ovarian syndrome (PCOS), one of the most common female endocrine disorders [23], has been suggested to arise through a gene–environment interaction, probably in the developmental milieu within which female gametes are formed [24]. Although maternal malnutrition is a major factor that adversely affects fetal growth and is associated with to lifelong consequences, relatively few studies have investigated the effects of MFR on the reproductive outcomes of offspring [4, 25, 26]. In a large epidemiological study women born to mothers exposed to famine were more reproductively successful compared to controls [27]. Moreover, malnutrition during pregnancy alters reproduction in sheep by inducing poor oocyte quality, may cause reproductive disruptions in rats mainly by an early vaginal opening, and induce a decrease in the primordial and antral follicle number [28–30]. During the first trimester of pregnancy in cattle, MFR leads to a reduced ovarian reserve in adulthood, as observed by the increased follicle stimulating hormone (FSH) levels [31].

At the moment, the available data regarding the relationship between the MFR and the female reproduction in the offspring have many limitations and gaps concerning the potential underlying mechanisms and almost all have evaluated the effects of maternal caloric restriction on the first generation only. Based on these considerations, the aim of the present experimental study was to investigate the impact of MFR in rats on ovarian architecture and function in first and second generations of female offspring.

## Methods

### Experimental design

First- and second-generation offspring [4, 32] of pregnant rat exposed directly (or indirectly through germline-independent transmission) to MFR were studied. Specifically, adult virgin Wistar female rats weighing 230 ± 20 g obtained from the Animal Unit at King Saud University were given free access to tap water and were fed *ad libitum* on standard rodent chow (23% protein, 4.5% fat, 3030 kcal/kg; lab diet 5001, Brentwood, MO). After being maintained in separate cages for four days of adaptation, they were mated with virgin adult males, and then were randomly divided into two groups: control group (group C, n. 15) received *ad libitum* food*,* and food-restricted group (group FR, n. 20) received only 50% of *ad libitum* food throughout gestation. The first generation of offspring (FR1) were fed *ad libitum*. After complete weaning, FR1 and control females were sacrificed before puberty (week 4, n. 10) and at adulthood (week 8, n. 10). The ovaries were removed and the fat was discarded. They were weighed using a digital balance (0.0001 g) and immediately fixed in 10% neutral buffered formalin at room temperature for classical histology. The remainder of FR1 females were allowed to reach sexual maturity, and were treated exactly as their mothers (FR females), i.e. 50% *ad libitum* food throughout gestation.

After birth, a second generation of the doubly food-restricted group females (FR2) was obtained. The FR2 offspring females were humanely sacrificed at 4 and 8 weeks, and their ovaries were collected, weighed and fixed exactly as before detailed for FR1 offspring females.

### Light microscopy

Ovaries were fixed in neutral buffer formalin (NBF) or Bouin’s fluid, and were subsequently preserved in 70% alcohol. At least three ovaries from each group have been cut in serial sections to a thickness of 7 μm using a Reichert-Jung microtome. Hematoxylin and eosin (H&E) staining was used to assess ovarian architecture in the group C, FR1, and FR2.

The effects of nutrition on folliculogenesis were evaluated by counting the number of primordial, primary, secondary, pre-antral, and antral follicles with visible oocyte nuclei in some slides for each ovary (see below). Specifically, ovarian follicles were classified according to the scheme of Pedersen and Peters (1968), with modifications. Primordial follicles included oocytes surrounded by a single layer of three to six squamous epithelial cells, whereas primary follicles were composed of oocytes surrounded by one layer of numerous cuboidal epithelial cells. Secondary follicles were characterized by oocytes surrounded by more than one layer of granulosa cells with no visible antral spaces. Antral follicles were composed of an oocyte surrounded by many layers of cuboidal granulosa cells, with many visible small antral spaces, or one large antrum. The theca layers and cumulus oophorus may be evident.

A particular interest was given to the occurrence of follicles containing multi-nuclei oocytes (MNOFs), key indicator of perturbations during germ cell nest formation [33–38]. The total number of multi-oocyte follicles (MOFs) was also counted in every section of the ovaries from the different groups.

To estimate the number of slides to be read for each ovary we used sample size calculations using the following formula [39]: $n\cong {z}_{1\hbox{-} \alpha /2}^2\frac{s^2}{h^2}$ where: s = sample standard deviation from an initial number (n_0_) of replications (n_0_ = 11), Z_1 − α/2_ = the value retrieved from the normal standard distribution, corresponding to the 1-α/2 probability (we choose α = 0.05) and h: the half width of the confidence interval.

### Statistical analysis

For data analysis of follicle number, we used the GraphPad prism version 5. Statistical comparisons were made using a two-tailed *t*-test. All values are presented as the mean ± standard deviation (SD). Significance was set at *P* value less than 0.05.

## Results

### Ovary weight

**Fig. 1:**
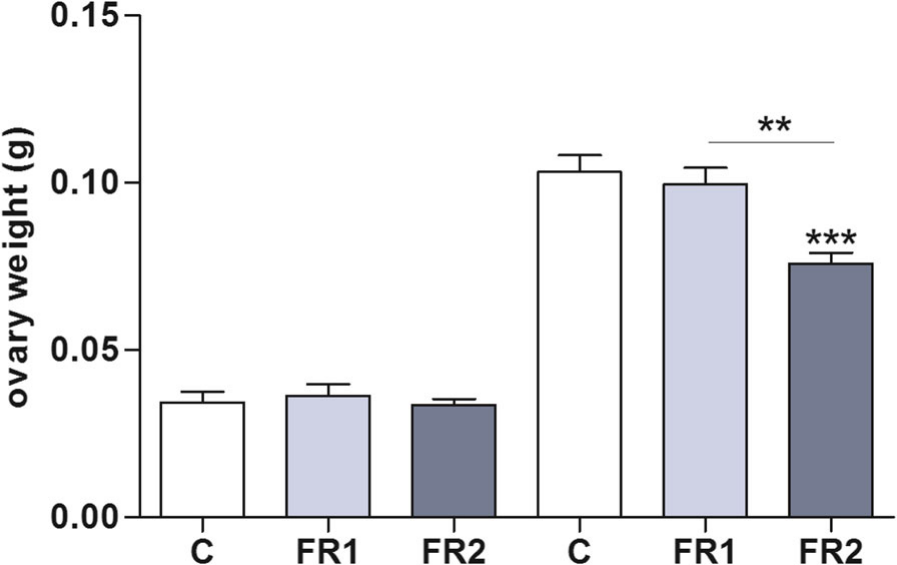
Effect of MFR on ovarian weight results in a significant reduction in 8-week-old FR2 females when compared to controls (*P* = 0.0003), and to FR1 females (*P* = 0.0011); no effect is observed in 4-week-old females

No difference in mean ovary weight was detected between FR1 and FR2 females vs. controls in 4-week-old. Conversely, in 8-week-old animals a significant (*P* = 0.0003) difference between intervention and control group in ovarian weight for FR2 females only (Fig. 1). A significant (*P* = 0.0011) difference was also observed in ovary weight in FR2 vs. FR1 females at week 8 (Fig. 1).

### Number of follicles

#### Total follicles

**Fig. 2:**
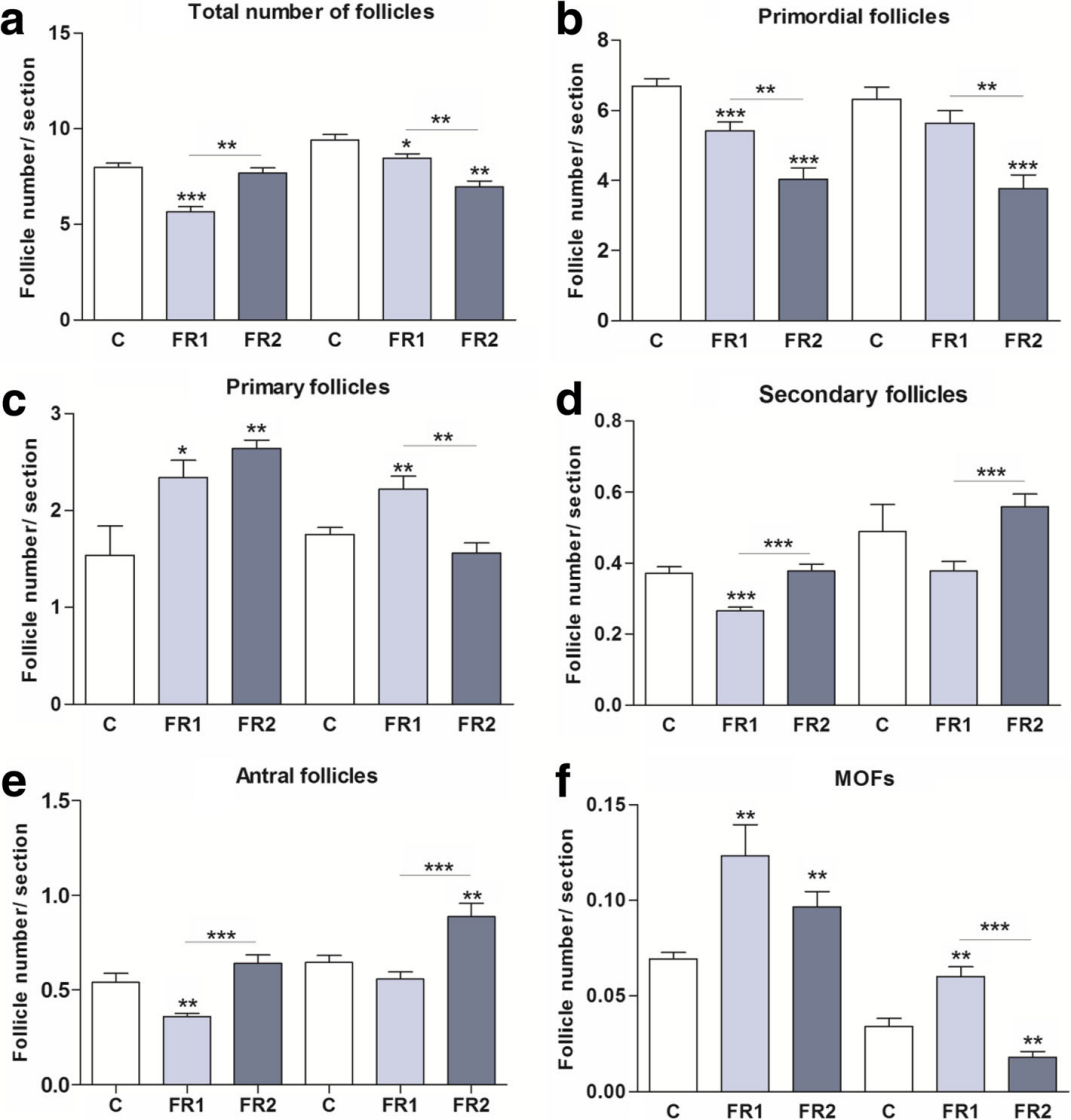
Effect of MFR on the number of follicles per section of ovarian tissue. **a** Total number of follicles: MFR significantly affects the total number of follicles in ovaries from FR1 females at 4 weeks (*P* = 0.0006) and 8 weeks (*P* = 0.0485), and from FR2 females at 8 weeks (*P* = 0.0013). The total number of follicles from FR2 at week 4 is higher vs. FR females (*P* = 0.0020), whereas it is lower in FR2 females at week 8 compared to FR females (*P* = 0.0074). **b** Primordial follicles: significant effect of MFR on the number of primordial follicles in FR1 and FR2 females at week 4 when compared to control (*P* = 0.0014 and *P* < 0.0001, respectively) and in FR2 females at week 8 (*P* = 0.0002). The total number of primary follicles from FR2 at both week 4 and 8 is significantly lower when compared to FR1 females (*P* = 0.0020 and *P = 0.003*, respectively). **c** Primary follicles are significantly higher in number in ovaries of 4-week-old females in both FR1 and FR2 vs. controls (*P* = 0.034 and *P =* 0.003). At week 8, FR1 females have significantly higher number of primary follicles vs. control (*P* = 0.0072), whereas FR2 females have significantly lower number of primary follicles vs. FR1 (*P* = 0.0014). **d** Secondary follicles: FR1 females at week 4 have significantly lower number of secondary follicles vs. control (*P* = 0.0002) and vs. FR2 (*P* = 0.001), whereas at week 8 FR2 have significantly higher number of secondary follicles vs. FR1 females (*P* = 0.0002). **e** Antral follicles: MFR has the same effect as in secondary follicles, except for a significant increase in FR2 females vs. control at week 8 (*P* = 0.008). **f** MOFs: The number of MOFs is significantly higher in ovaries from FR and FR2 females vs. control at week 4 (*P* = 0.0044 and *P =* 0.006, respectively); it is also significantly higher in FR1 females at week 8 (*P* = 0.0013), and again significantly lower in ovaries from FR2 females vs. control (*P* = 0.0075). FR2 females at week 8 have significantly lower number of MOFs when compared to FR1 females (*P* < 0.0001). All results are given as mean ± SD; *P* < 0.05, *FR1 vs. controls (C) and FR2 vs. controls, * with error bars: FR1 vs. FR2

Total number of ovarian follicles was significantly (*P* = 0.0006) lower in FR1 females at week 4 in comparison with controls, while it was not different in FR2 females when compared to the controls (Fig. 2a). At week 8, the total number of follicles in ovaries from both FR1 and FR2 females resulted significantly (*P* = 0.0485 and *P =* 0.0013, respectively) lower than in controls. The total number of follicles was significantly (*P* = 0.0020 and *P* = 0.0074, respectively) higher and lower in FR2 vs. FR1 females at week 4 and 8, respectively.

#### Primordial follicles

The amount of the primordial follicle was significantly lower in FR1 and FR2 females than in controls at both week 4 (*P* = 0.0014 and *P* < 0.0001, respectively), and at week 8 (*P* = 0.0002 and *P* = 0.0044, respectively) (Fig. 2b).

#### Primary follicles

The number of primary follicles was significantly (*P* = 0.034 and 0.003, respectively) higher in FR1 and FR2 vs. controls at week 4. That statistical (*P* = 0.0072) differences were sustained at week 8 only in FR1 females vs. controls (Fig. 2c). A significant (*P* = 0.0014) difference in the number of primary follicles was observed in FR2 vs. FR1 females at week 8.

#### Secondary follicles

The number of secondary follicles was significantly (*P* = 0.0002) lower in the ovaries of FR1 females compared to controls only at week 4 (Fig. 2d). Both at week 4 and 8, the number of secondary follicles was significantly (*P* = 0.0010 and *P* = 0.0002, respectively) higher in FR2 than in FR1 females.

#### Antral follicles

When compared to controls, the number of antral follicles was significantly lower (*P* = 0.0023) in the ovaries of FR1 females at week 4, and significantly higher (*P* = 0.0084) in the ovaries of FR2 females at week 8 (Fig. 2e). A significant (*P* = 0.0008 and *P* < 0.0001, respectively) difference in the number of antral follicles was detected between FR2 and FR1 females at week 4 and 8.

### Ovarian histopathology

#### MOFs

**Fig. 3:**
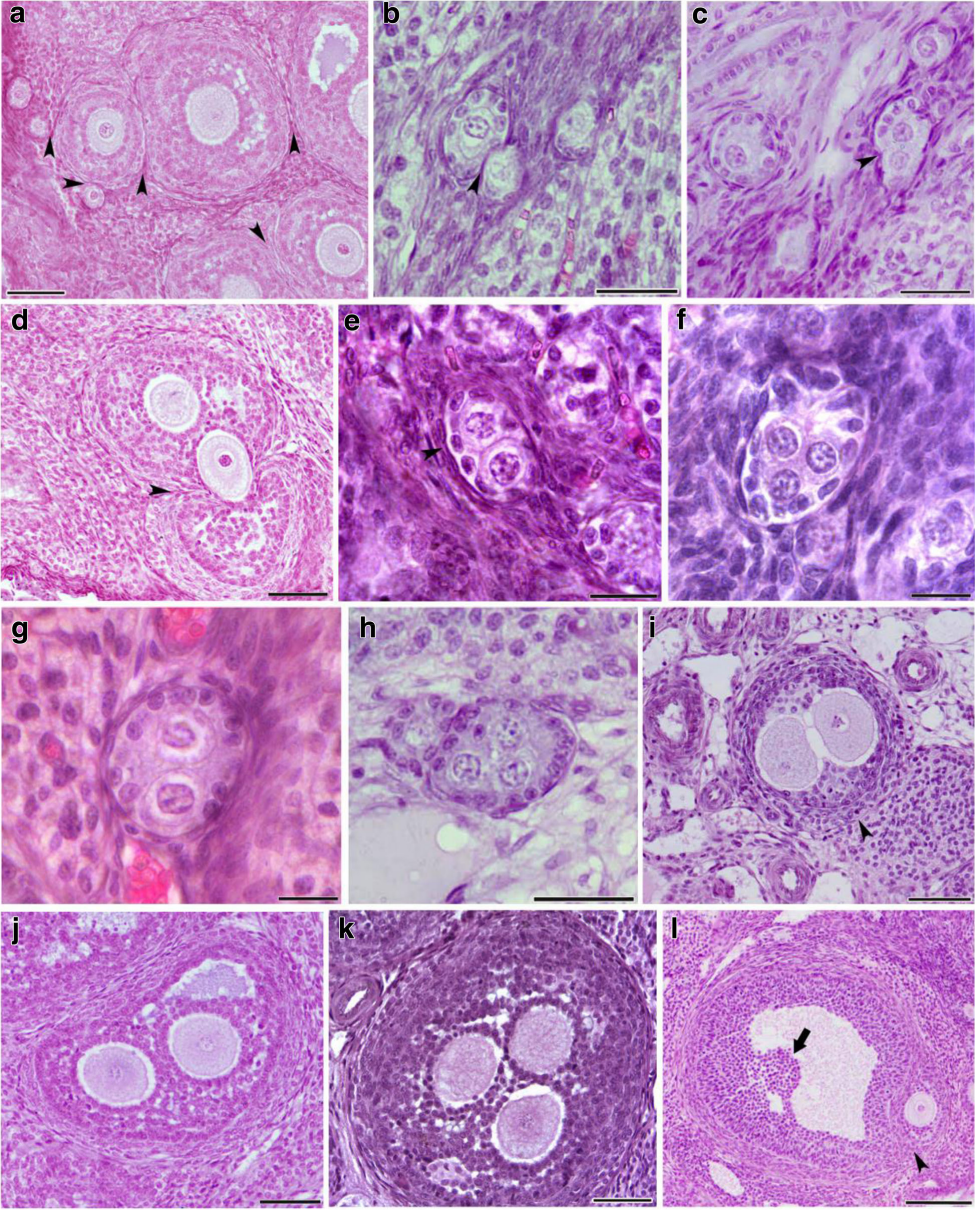
Hematoxylin and eosin staining of paraffinized ovarian sections from FR1 and FR2 females showing the generation of MOFs. **a** Ovaries from FR1 and FR2 females are mainly characterized by many growing follicles that are adjacent to each other, indicating their fusion. **b-d** Follicles in the process of merging (arrowheads); we can see in (**d**) the displacement of the oocyte from one follicle into the second one. **e** Primordial follicle with two oocytes. **f** primordial follicle with three oocytes. **g** Primary follicle with two oocytes, (**h**) Primary follicle with three oocytes. **i** Secondary follicle with two oocytes. **(J)** Antral follicle with two oocytes; (**k**) Antral follicle with three oocytes; (**l**) Antral follicle merging with an early secondary follicle (arrowhead), the large arrow is showing the oocyte position of the antral follicle. Scale bar = 200 μm

MOFs population was found in all the studied groups and at all follicular stages, from the primordial to the large antral stage; these MOFs contained two or more oocytes (Fig. 3a-f). The architecture of the ovaries from FR1 and FR2 females was mainly characterized by more growing follicles when compared to controls. In most of the cases, these were adjacent to each other, suggesting that a fusion has occurred (Fig. 3a). Furthermore, we even reported joining follicles, characterized by the displacement of the oocyte from one follicle into another (Fig. 3b).

When compared to the controls, the MOFs were significantly (*P* = 0.0044 and *P =* 0.006, respectively) more frequent in ovaries from FR1 and FR2 females at week 4 (Fig. 2f). Nevertheless, while this number was significantly higher (*P* = 0.0013) in FR1 females compared to control at week 8, it was significantly (*P* = 0.0075) lower in ovaries from FR2 females.

#### MNOFs (Heterokaryon)

**Fig. 4:**
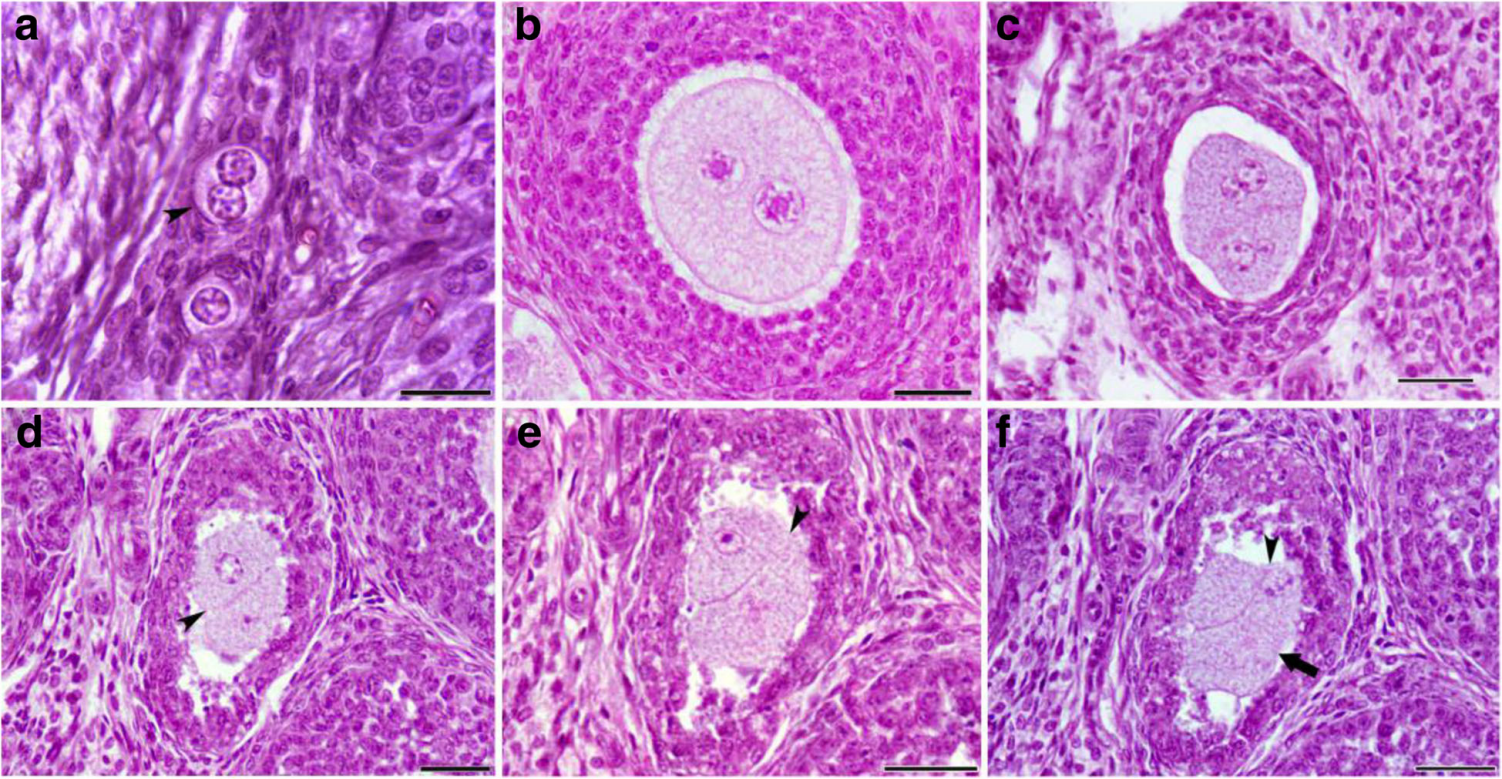
Hematoxylin and eosin staining of paraffinized ovarian sections from FR1 and FR2 females showing the generation of MNOFs. **a** Primordial follicle with two nuclei- oocyte. **b** Secondary follicle containing one oocyte with two proportional nuclei (**c**) Secondary follicle containing one merged oocyte with four disproportional nuclei. **d** Secondary follicle with two oocytes that are very close to each other, suggesting that they will probably fuse soon to form a heterokaryon. **e** Secondary follicle containing two semi-fused oocytes (arrowhead). **f** The same secondary follicle in (**e**) but at another level of section. Scale bar = 200 μm

A high frequency of MNOFs was detected in the ovaries of FR1 and FR2 females compared to controls (Fig. 4a-b). A detailed analysis of these MNOFs provided clues on how they were generated. Specifically, we detected many MOFs in which oocytes were frequently observed very close to each other (Fig. 4d), suggesting they had fused to form a heterokaryon. In some cases, oocytes within the same follicle were apparently undergoing such a joining process (Fig. 4e).

## Discussion

The fertile reproductive lifespan of female mammals is mainly linked to the initial ovarian reserve of primordial follicles that reaches its maximum level around the time of birth, and is gradually depleted during reproductive life [40–42]. At prepubertal age (week 4), we found that the number of primordial follicles was significantly decreased in FR1 and FR2 rats, suggesting that MFR affects the ovarian reserve of primordial follicles during early fetal life. The significant decrease in the number of primordial follicles at week 4 was associated with an increase in the number of primary follicles among the different studied groups suggesting that MFR might cause an early menarche by inducing early folliculogenesis. This hypothesis seems consistent with previous studies [26, 43].

At week 8, the significant decrease in ovarian weight related to the total number of follicles in both first and second generation offspring after MFR is mainly caused by the significant decrease in primordial follicles in the ovarian reserve compared to controls. This could suggest an early menopause in FR2, which is less likely in FR1 animals. In fact, previous studies reported that MFR is associated with early menarche and menopause [26, 43–45]. To this regard, it is possible to hypothesize that MFR provided first and second generation offspring with a phenotype that is better suited for the lack of food. That new phenotype is consistent with the trade-off theory, i.e. an increase in fertility and a decrease in reproductive lifespan may lead to an increase in the chances of an organism to reproduce successfully [46]. In fact, fetal growth restriction can be considered as a part of the life history strategy for FR1 and FR2 females that were in utero when their mothers underwent food restriction. Since prenatal undernutrition leads to reduced longevity in mice [47], these females may anticipate a shorter life because of a higher risk of extrinsic mortality. It is possible to hypothesize that they may have to adjust their reproductive aptitude by changing the intensity and duration of their lifespan, the timing of the stages of folliculogenesis, as well as the age at which they should reach reproductive maturity. Due to the lack of food sensed through nutritional or endocrine signaling during fetal life [48], and to ensure reproductive success before death, these females have probably programmed their reproductive lifespan to be very intensive but relatively limited in time, which is consistent with population regulation in the theory of life history [49]. Thus, when they reach prepubertal age, they may upregulate the expression of genes involved in steroidogenesis, which in turn induces folliculogenesis for a greater number of primordial follicles while concomitantly explaining the significant higher number of in primary follicles in 4-week-old FR1 and FR2 females vs. control. The relatively large number of induced follicles undergoing folliculogenesis at one time makes follicles adjacent to each other and, consequently, highly increases the probability that they will merge to form MOFs [34]. This may have decreased the number of growing oocytes (secondary and antral follicles), and may explain the lower total number of follicles, and the higher number of MOFs in 4-week-old FR1 females (vs. controls). This strategy is also associated with a faster decline in ovarian function with aging, that is clearly supported by the significant decrease in the number of primordial follicles in the ovarian reserve, and also by the significant decrease in the total number of follicles in both FR1 and FR2 females at an early age (8-week), that corresponds in normal females to a high reproductive performance.

Of note, a higher number of MOFs in ovaries from 4-week-old first and second generation females was observed. Many follicles at these stages may have fused to form MOFs, which explains their significant higher number in FR1 females at four weeks, while this is less likely in FR2 females. While the mechanism of MOFs formation during nest breakdown has been described [33, 50], this is the second study that clearly confirms a new mechanism for the generation of MOFs through the fusion of follicles in the mammalian ovary, and their incidence increased sharply at prepubertal age [34]. Inversely, we found that the number of secondary and antral follicles was significantly lower in FR1 and FR2 at prepubertal age when compared to controls. Previous findings have reported that most cases of MOFs represent a fusion between secondary follicles, or a fusion between one secondary and one large antral follicle [34]. Furthermore, the number of secondary/antral follicles observed in 8-week-old females was higher than in controls when the number of MOFs was lower. This result is also consistent with the finding of Perez-Sanz and co-workers that showed that the number of MOFs declines significantly in female mice when they become sexually mature [33].

The presence of MNOFs is exceptional and represents a challenge for future studies. Two different mechanisms could explain the origin of this phenomenon: the nest breakdown–follicle assembly, and the fusion of more than one oocyte within the same multi-oocyte follicle. Based on our findings, the second mechanism appears the most probable since it is suggested that MOFs are most likely generated by the assembly of follicles [34] rather than being produced early on during the formation of the ovary. The presence of more than one oocyte in direct contact within the same follicle highly increases the possibility of their fusion, and leads to the formation of MNOFs. In fact, any cell brought into contact with another cell and given the right conditions (such as sufficient amounts of fusogen proteins, simultaneously present on each of the two cell surfaces) will fuse with the second cell, even when the latter is foreign [51–53]. This fusion can be beneficial mainly during embryonic development, and for cell-based therapies, and represents a well-known process during reproduction when gametes (spermatozoa and oocytes) unite during fertilization to form the zygote. It has also been described in muscle cells, macrophages and nerve cells [54–58]. When cell fusion is blocked during embryonic development, defects in organogenesis, embryonic lethality, and postembryonic defects can increase [53, 59] suggesting that fused cells are hybrid cells or chimera that function efficiently during embryonic development, driving correct organ formation [54]. In *Caenorhabditis elegans*, particularly in the proliferative zone of germ cells, two or more crescent shaped nuclei have been observed [60]. MNOFs have also been described during the initial stages of oogenesis in some amphibian species. For example, in *Ascaphus truei,* oogenesis involves eight nuclei [61], whereas frog oocytes with two nuclei have been described in *Leiopelma hochstetteri* [62]. The most evident example of the necessity for multi-nucleated cells is the syncytiotrophoblast, which is formed when embryonic cytotrophoblast cells fuse with the maternal endometrial epithelium [63, 64]. These cells represent the most important cell type in the placenta, and there is a strong correlation between a successful pregnancy and healthy syncytiotrophoblasts, likely due to their multi-nuclear state [64].

## Conclusions

Current data suggest that MFR influences ovarian histopathology and, in turn, the reproductive health of first and second generation female offspring during fetal development, and they have been probably programmed to have an early menarche by inducing early folliculogenesis, and an early decline in ovarian function thereby decreasing the reproductive lifespan, and leading to an early menopause.

## Abbreviations

FR1: Female rat offspring from the first generation
FR2: Female rat offspring from the second generation
FSH: Follicle stimulating hormone
H&E: Hematoxylin and eosin
MFR: Maternal food restriction
MNOFs: Follicles containing multi-nuclei oocytes
MOFs: Multi-oocyte follicles
NBF: Neutral buffer formalin
SD: Standard deviation
